# Circulating eosinophil progenitors express major trafficking related molecules and are more activated compared to mature eosinophils in patients with asthma

**DOI:** 10.1186/2045-7022-3-S1-P7

**Published:** 2013-05-03

**Authors:** You Lu, Carina Malmhäll, Margareta Sjöstrand, Madeleine Rådinger, Bo Lundbäck, Jan Lötvall, Apostolos Bossios

**Affiliations:** 1 University of Gothenburg, Sahlgrenska Academy, Institute of Medicine, Krefting Research Centre, Sweden

## Background

Eosinophilic inflammation represents a hallmark for allergic asthma. Eosinophils differentiate in the bone marrow from CD34+ cells and are released into the blood and traffic to the lung tissue. To date, the majority of studies originate from animal models or from humans after allergen exposure. Thus, it is unclear if mature eosinophils and eosinophil progenitors express similar levels of trafficking related molecules. Therefore, we characterized the expression of trafficking related molecules on circulating eosinophil progenitors in patients with stable asthma.

## Methods

Participants, 13 patients with stable asthma; 7 at the high end (≥0.3x10^9^/l) and 6 at the low end (≤0.2x10^9^/l) of normal range of blood eosinophils, and 5 healthy controls were selected from the West Sweden Asthma Study. Airway eosinophils were studied in induced sputum. Mature (CD45+IL-5Rα+SSC^high^) and progenitors (CD45+CD34+IL-5Rα+SSC^low^) eosinophils and their expression of selectin (PSGL-1), integrins (VLA-4:CD49d+CD29+, Mac-1:CD11b+CD18+), eotaxin(s) receptor (CCR3+), activation (CD69+, CD25+) and apoptosis (active-caspase 3, CD95+) were quantified in fresh blood by flow cytometry.

## Results

Asthma patients with high blood eosinophils had increased sputum eosinophils and blood eosinophil progenitors compared to the healthy controls (p<0.05). Mature eosinophils and eosinophil progenitors expressed similar levels of PSGL-1 and VLA-4. Mac-1 was expressed in all mature eosinophils but was reduced in progenitors, in all groups (<0.01). Mature eosinophils expressed higher levels of CCR3 compared to progenitors (p<0.05). However, the CCR3+ eosinophil progenitors showed increased expression of CD25 and CD69 i.e. were more activated in all groups (<0.01) compared to mature eosinophils. No differences in the expression of apoptosis related markers were found between CCR3+ eosinophil progenitors and CCR3+ mature eosinophils.

## Conclusion

Circulating eosinophil progenitors in patients with stable asthma express major trafficking related receptors found in mature eosinophils, thus suggest their capacity for migrating to the lung tissue. Notably, the eosinophil progenitors primed to eotaxin(s) (CCR3+) are highly activated compared to mature eosinophils.

